# Transfer of a rational formulation and process development approach for 2D inks for pharmaceutical 2D and 3D printing

**DOI:** 10.1016/j.ijpx.2024.100256

**Published:** 2024-05-10

**Authors:** Maximilian Schulz, Malte Bogdahn, Simon Geissler, Julian Quodbach

**Affiliations:** a Institute of Pharmaceutics and Biopharmaceutics, Heinrich Heine University Düsseldorf, Universitätsstr. 1, Düsseldorf, Germany; b Merck Healthcare KGaA, Frankfurter Str. 250, Darmstadt, Germany; c Division of Pharmaceutics, Utrecht Institute for Pharmaceutical Sciences, Utrecht University,Universiteitsweg, 99, Utrecht, the Netherlands

**Keywords:** Inkjet Printing, 2d printing, Binder jetting, Jettable window, Z-number

## Abstract

The field of pharmaceutical 3D printing is growing over the past year, with Spitam® as the first 3D printed dosage form on the market. Showing the suitability of a binder jetting process for dosage forms. Although the development of inks for pharmaceutical field is more trail and error based, focusing on the *Z*-number as key parameter to judge the printability of an ink. To generate a more knowledgeable based ink development an approach from electronics printing was transferred to the field of pharmaceutical binder jetting. Therefore, a dimensionless space was used to investigate the limits of printability for the used Spectra S Class SL-128 piezo print head using solvent based inks. The jettability of inks could now be judged based on the capillary and weber number. Addition of different polymers into the ink narrowed the printable space and showed, that the ink development purely based on *Z*-numbers is not suitable to predict printability. Two possible ink candidates were developed based on the droplet momentum which showed huge differences in process stability, indicating that the used polymer type and concentration has a high influence on printability and process stability. Based on the study a more knowledgeable based ink design for the field of pharmaceutical binder jetting is proposed, to shift the ink design to a more knowledgeable based and process-oriented approach.

## Graphical abstract

**Figure:**
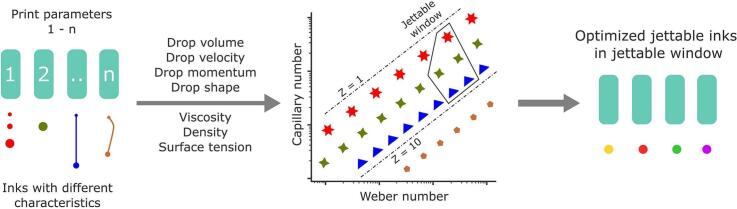

## 1 Introduction

Printing technologies are attracting more and more interest in the pharmaceutical field within the recent years. They have emerged as the most promising technology platform for the implementation of personalized medicine (Park et al., 2018; Daly et al., 2015). Inkjet or 2D printing involve the spatial deposition of a liquid ink, either a solution or a suspension, out of a print head. 3D printing, also known as additive manufacturing, is a method of building up a 3D object by fusion or deposition of material in a layer-by-layer process such as fused deposition modeling (FDM), selective laser sintering (SLS), stereolithography (SLA), semisolid extrusion or binder jet printing (Vaz and Kumar, 2021). For this process, the desired structure is modeled using a computer-aided design (CAD) software and these designs are then transformed into stl-files, which can be interpreted and read by a machine and transferred into a layer-by-layer deposition of material (Park et al., 2018; Jacob et al., 2020). Thereby objects with various forms and geometries can be produced, enabling the possibility to achieve variable dosages, complex drug release profiles, and, thus, personalization (Al-Metwali and Mulla, 2017; Infanger et al., 2019; Kyobula et al., 2017; Wang et al., 2006).

2D and 3D printing of personalized medicine is still heavily investigated and translation to applications in (hospital and community) pharmacies is still in its infancy. 3D printing was successfully used for initial stages of drug development, especially for pre-clinical studies and has a high potential for first-inhuman trials, as it shows an excellent dose flexibility and dosage forms can be produced on site (Awad et al., 2018; Matic et al., 2020; Trenfield et al., 2018). It has already been established commercially within the pharmaceutical field as in August 2015, the first 3D printed tablet, Spritam®, was approved by the FDA (U.S. Food and Drug Administration, Center for Drug Evaluation and Research, 2024). Spritam® is produced by binder jetting, making it the first FDA approved 3D printing technology for oral dosage forms (U.S. Food and Drug Administration, Center for Drug Evaluation and Research, 2024). Binder jetting can be regarded as a combination of 2D and 3D printing. A solvent or an adhesive liquid is jetted onto a powder layer, which either binds the powder due to adhesive forces or dissolves a binder in the powder. After the powder layer has partially dried, this process is repeated until the final object is built. This has the advantage that very high drug loadings can be achieved (Infanger et al., 2019). Compared to traditional tablet production, less excipients are necessary. In the most simple case, only a powdered drug and a binder liquid are necessary. Binder jetting generates tablets with comparably low tensile strength and higher friability but rapid disintegration, as powder is only loosely bound via adhesive forces of the binder or solid bridges (Infanger et al., 2019). Researchers also utilized other inks for 3D manufacturing of drug products, such as photo-curable inks (Clark et al., 2020) or waxes (Wang et al., 2004).

The inkjet technology used in binder jetting can be divided into two categories: continuous jet (CJ) and Drop on Demand (DoD) printing. In CJ printing, a continuous stream of liquid is generated, which is fast but has low accuracy. In DoD printing, individual droplets are generated, resulting in better process control. For droplet generation, either a thermal or piezoelectric printhead is used. Thermal printheads are not commonly used in the pharmaceutical field, as researchers have brought up that the high process temperatures might influence thermal sensitive APIs (Afsana, Jain, V., Haider, N., Jain, K, 2018; Bansal et al., 2018; Warsi et al., 2018; Shirazi et al., 2015). It is still unclear and discussed in the community weather the short rise in temperature, lasting only few milliseconds, degrades the API and comparably little research is performed using thermal printheads (Montenegro-Nicolini et al., 2024; Buanz, and Saunders Mh Fau - Basit, A. W.; Basit Aw Fau - Gaisford, S.; Gaisford, S, 2024; Meléndez, and Kane Km Fau - Ashvar, C. S.; Ashvar Cs Fau - Albrecht, M.; Albrecht M Fau - Smith, P. A.; Smith, P. A, 2024; Vuddanda et al., 2024). In piezoelectric printheads, the piezoelectric element within the printhead is excited with a certain voltage, creating a deformation wave within the liquid, pushing a specific amount of ink through the nozzle. Droplet generation is mainly driven by surface tension and inertial forces, which are created when the droplet is separated from the liquid. Remaining liquid in the printhead travels back into the printhead chamber, as the relaxation of the piezo element leads to a pressure drop within the printhead chamber (Afsana, Jain, V., Haider, N., Jain, K, 2018; Derby, 2010; Guo et al., 2017). The droplet formation and ejection behavior is primarily dependent on the viscosity, surface tension and inertial forces of the liquid. To describe the printability of an ink, different dimensionless numbers are frequently used (Fromm, 1984). The Ohnesorge (Oh) number (Eq. 1), which is a ratio of the Weber (*We*) number (Eq. 2) and Reynolds (*Re*) number (Eq. 3) describes the droplet formation, while mainly considering the viscous forces. The *We* number describes the droplet formation in relation to the surface tension and the inertial forces of the liquid while the *Re* number considers the effect of the inertial forces and viscous forces on droplet formation. Further, the capillary (Ca) number (Eq. 4) is used to describe the relation between surface tension and viscous force (Sen et al., 2020; Nallan et al., 2014).

(1) $$\;\mathrm{Oh}=\frac{\mathrm{viscous forces}}{\sqrt{\text{inertial force}*\text{surface tension}}}=\frac{\mathrm{\eta \nu d}}{\sqrt{\rho \nu 2d3\sigma }}=\frac{\eta }{\sqrt{\mathrm{\rho \sigma d}}}$$

(2) $$\mathrm{We}=\frac{\mathrm{inertial force}}{\mathrm{surface tension}}=\frac{\rho \nu 2d2}{\mathrm{\sigma d}}=\frac{\rho \nu 2}{\sigma }$$

(3) Re=inertial forceviscous force=ρν2d2ηνd=ρνdη

(4) $$\;\mathrm{Ca}=\frac{\mathrm{viscous force}}{\mathrm{surface tension}}=\frac{\mathrm{\eta \nu d}}{\mathrm{\sigma d}}=\frac{\eta \nu }{\sigma }$$

d and *ν* are the nozzle diameter and droplet velocity and ρ, σ and *η* are the density, surface tension and viscosity of the ink. In literature, the inverse Oh number, also known as *Z*-number (Eq. 5), is used to describe the printability of an ink. It governs the most relevant parameters of the liquid, the inertial forces, surface tension and viscous forces, as the ratio of the *Re* number to the square root of the *We* number.

(5) $$\;Z=\frac{1}{\mathrm{Oh}}=\frac{\sqrt{\mathrm{inertial force}*\mathrm{surface tension}}}{\mathrm{viscous force}}=\frac{\sqrt{\rho \nu 2d3\sigma }}{\mathrm{\eta \nu d}}=\frac{\sqrt{\mathrm{\rho \sigma d}}}{\eta }$$

This dimensionless *Z*-number was introduced by Fromm and is since commonly used to describe the jettability of liquids for printing processes (Fromm, 1984). The jettability is the property of an ink to form stable droplets suitable for the respective printing process. Fromm concluded that fluids with a *Z*-number lower than 2 are not capable of forming stable droplets. Still, in literature, inks with a Z-number between 1 and 10 are generally considered as jettable (Reis and Derby, 2011) but the range varies across different publications (Nallan et al., 2014; Jang et al., 2009; Kholghi Eshkalak et al., 2017; Holland et al., 2018; Wilts et al., 2019; Hoeng et al., 2017; Liu and Derby, 2019). This variability shows that using only the *Z*-number to describe jettability of an ink is not sufficient.

Presently, the development of an ink for 2D printing within the pharmaceutical field is rather an art than a knowledge-based process. In the last years, researcher started to use machine learning to predict the jetting behavior of inks in the field of ink jetting and pharmaceutical ink jetting and to design process parameters or monitor the process (Kim, S.; Cho, M.; Jung, S, 2024; Phung et al., 2024; Queraltó et al., 2024; Zhang and Moon, 2024). But often inks are still designed using the specifications for ink surface tension, viscosity, and density of the print head manufacturer and are adjusted slightly to achieve printability (Clark et al., 2020; Clark et al., 2017; Cader et al., 2019; Alomari et al., 2018; Acosta-Velez et al., 2018a; Acosta-Velez et al., 2017; Acosta-Velez et al., 2018b; Wickstrom et al., 2017; Thabet et al., 2018; Trenfield et al., 2019). A more systematic approach was proposed by Infanger et al., which includes a cycle of adjusting the inks to reach the desired quality attributes (Infanger et al., 2019). But these approaches lack the understanding whether a modification of viscosity, surface tension, or density will lead to a better printability.

**Fig. 1:**
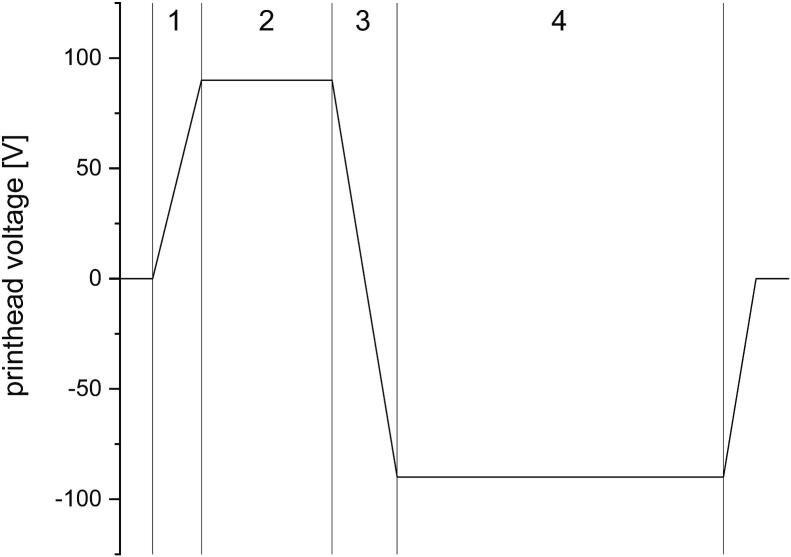
Schematic of a typical piezo excitation waveform. 1) rise time, 2) dwell time, 3) fall time, and 4) echo time.

**Fig. 2:**
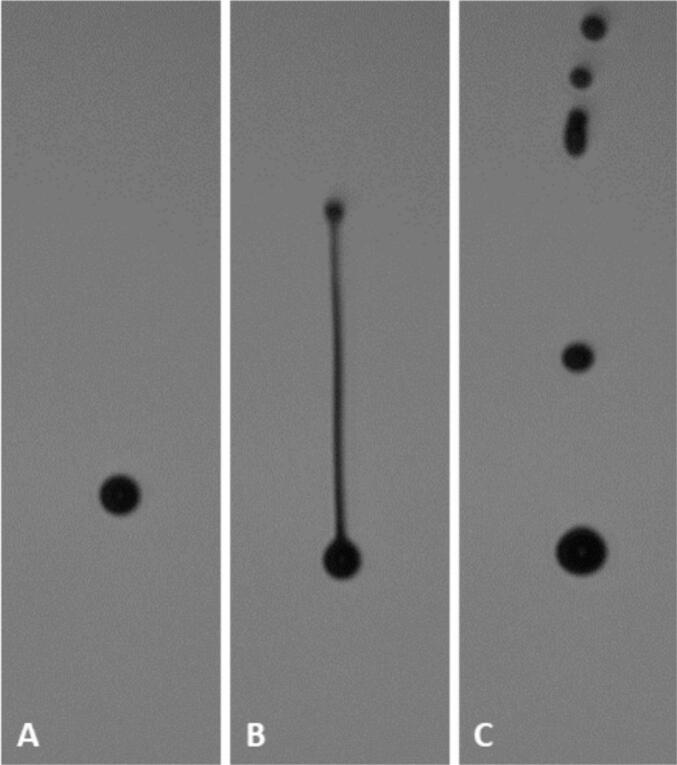
Different droplet formation processes. A) single droplet, B) droplet with ligament, C) droplet with multiple satellite droplets.

**Fig. 3:**
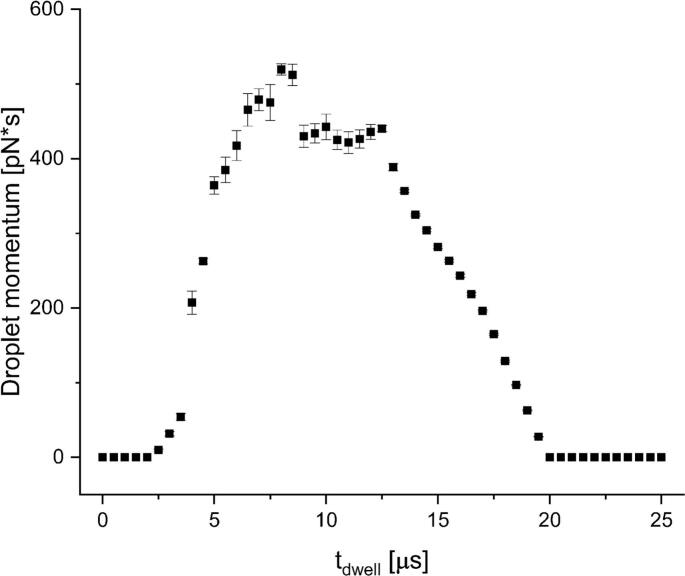
Optimal dwell time determination exemplarily shown for glycerol 75%; the optimal dwell time was determined as 8.0 μs. (mean ± SD *n* = 9).

**Fig. 4:**
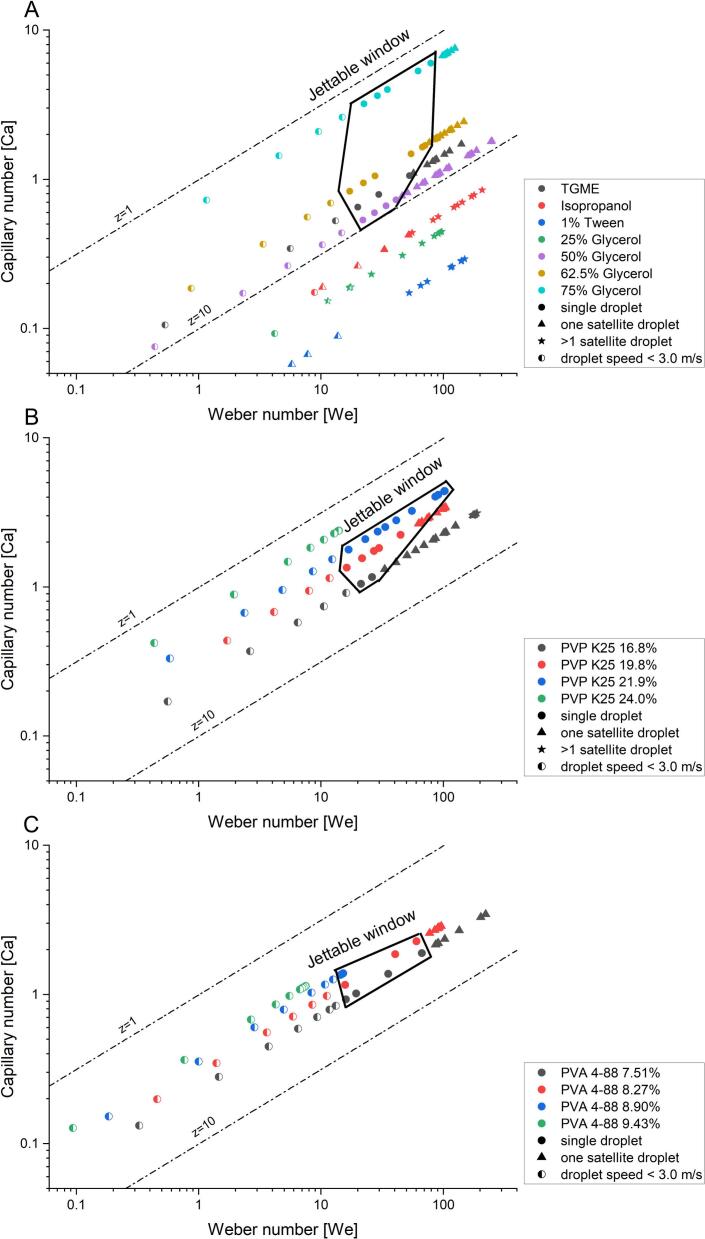
Jettable windows of (A) selected solvents and solvents mixtures, (B) inks containing PVP and (C) inks containing PVA. Inks that form single droplets with a droplet velocity of at least 3.0 m/s within 1.3 mm from the nozzle tip are considered as jettable.

This problem was acknowledged by Nallan et al. for the development of nanoparticle-containing inks (Nallan et al., 2014). In their publication, they proposed a systematic ink design approach. Initially, the required characteristics of jettability are defined. This includes possible failure modes and the definitions of boundaries of the jettable space for the used system. To define the boundaries of the jettable space, droplet requirements have to be defined, e.g., are satellite droplets acceptable or not. Further a minimum or maximum droplet size should be defined. If needed for the application, the droplet speed after ejection can be a relevant parameter as well. The defined space can then be explored using solvents and solvents mixtures. For this, the excitation waveform of the piezo element needs to be optimized. The pressure wave propagation can vary significantly between different ink systems, which has an influence on droplet speed and volume (Nallan et al., 2014; Bogy and Talke, 1984). Therefore, the optimal excitation waveform (Fig. 1) has to be determined for each ink system. For the wave optimization, the rise and fall time (Fig. 1, Fig. 3) of the pulse should be kept as short as possible since longer ramp times can lead to large droplet volumes (Bogy and Talke, 1984). To generate a sufficient suppression of unwanted residual deformation waves after droplet ejection, it is recommended that the echo time (Fig. 1, Fig. 4) should be twice the dwell time (Fig. 1, Fig. 2) (Kye-Si, 2009). During the echo time, a negative voltage is applied to the piezo element to reduce the movement of the piezo element to a minimum, as it reverts the movement direction of the piezo element. The pulse shape of the piezo element can have significant effects on the satellite droplet formation (Kwon, 2009; Kim et al., 2022; Jianjun et al., 2023). Some researcher introduce multi-pulsed waveforms to reduce ligament and satellite droplet formation (Oktavianty et al., 2019; Shah et al., 2020). But the design and optimization of wave forms is still very time-consuming, despite the development of waveform design methodologies due to the limited knowledge of wave form propagation and drop formation.

The jettability and the system boundaries can then be investigated using a dimensionless space, e.g., the We-Re space as suggested by Derby et al (Derby and Reis, 2011).

This study has multiple aims. The first aim was to transfer the development approach of Nallan et al. to the field of pharmaceutical inkjet printing with the potential applications of 2D printing and 3D binder jetting to demonstrate a data driven and rational ink development process. The second aim was the expansion of the approach to binder (polymer) containing inks and to compare the resulting jettable windows. This should allow the prediction of jettability of binder containing inks by using the jettable window explored with solvent based inks for the given printhead. Lastly, to better understand additional important factors for the ink development, the printing process should be characterized and the jetting consistency during printing investigated. For this work, the Ca-We space was used, as proposed by Nallan et al (Nallan et al., 2014). This study only considers solution inks and, based on the assumption that dissolved APIs change important ink characteristics, such as surface tension and viscosity, less than added surfactants and polymer, no API was added to simplify the formulations.

## 2 Material and methods

### 2.1 Materials

For the investigation of the jettable windows, different solvents were used: tri(ethylene glycol) monoethyl ether (TGME, Sigma-Aldrich, USA), isopropanol (technical grade, Th. Geyer, Germany), an aqueous 1% (*w*/w) polysorbate 20 solution (Tween 20, Caesar & Loretz, Germany), and different glycerol solutions prepared from glycerol 85% (Ceasar & Loretz, Germany). Demineralized water was used for all water containing inks. The detailed compositions are shown in Table 1.

**Table 1:** Composition of solvent based inks.

Ink	Solvent Concentration [*w*/w %]	Water [w/w %]	Tween 20 [w/w %]
Glycerol 75%	75.0	24.0	1.0
Glycerol 62.5%	62.5	36.5	1.0
Glycerol 50%	50.0	49.0	1.0
Glycerol 25%	25.0	74.0	1.0
Tween 20	1.0	99.0	–
Isopropanol	100.0	–	–
TGME	100.0	–	–

The binder containing inks consisted of different aqueous solutions of either polyvinylpyrrolidone K25 (PVP K25, BASF, Germany), polyvinyl alcohol 4–88 (PVA Parteck® MXP, Merck, Germany) or polyvinyl alcohol 18–88 (PVA, Emprove® Essential, Merck, Germany) and 1% w/w Tween 20 for surface tension adjustment. The detailed compositions are shown in Table 2.

**Table 2:** Composition of polymer-containing inks.

Polymer	Polymer concentration [*w*/w %]	Water [w/w %]	Tween 20 [w/w %]
PVP K25	16.8	82.2	1.0
PVP K25	19.8	79.2	1.0
PVP K25	21.9	77.1	1.0
PVP K25	24.0	75.0	1.0
PVA 4–88	7.51	91.49	1.00
PVA 4–88	8.27	90.73	1.00
PVA 4–88	8.90	90.10	1.00
PVA 4–88	9.43	89.57	1.00
PVA 18–88	4.65	94.35	1.00
PVA 18–88	3.80	95.20	1.00
PVA 18–88	2.75	96.26	1.00

### 2.2 Methods

#### 2.2.1 Ink preparation

All inks were prepared with demineralized water and stirred for at least 12 h prior to use. PVA inks were additionally heated for two hours at 80 °C to ensure complete dissolution. All Inks were degassed for 30 min in an ultrasonic bath and filtered through a 0.45 μm hydrophilic polyamide membrane filter (Chromafil® Xtra PA-45/25, Macherey-Nagel) prior to use to remove particles that could block the nozzles.

#### 2.2.2 Ink characterization

##### 2.2.2.1 Dynamic viscosity

The dynamic viscosity of all solvents and inks was determined using a rotational viscosimeter (Kinexus pro, Netzsch, Germany). All measurements (*n* = 3, mean ± sd) were conducted using a 65 mm plate and 1°/60 mm cone with a 300 μm gap at 30.0 °C and at the highest possible shear rate of 1000 s^−1^. This shear rate was reported to occur within the print head (Cader et al., 2019; Hoath et al., 2014; Hoath, 2016). Samples were trimmed according to the Malvern user guide before each measurement.

##### 2.2.2.2 Surface Tension

Surface tension was determined using a Tensiometer K100 (Krüss, Germany) utilizing the Wilhelmy plate method. The surface tension was measured 10 times in 60 s and averaged. All measurements were conducted in triplicates at 30 °C.

##### 2.2.2.3 Density

The density of all solvents and inks was determined using a calibrated pycnometer at 30 °C. All inks and solvents were degassed in an ultra-sonic bath for 30 min at the highest intensity prior measurement (*n* = 3, mean ± sd).

#### 2.2.3 Binder jetting

A PIXDRO LP50 printer (Meyer Burger, The Netherlands) was used. All droplets were generated using a Spectra S Class SL-128 piezo print head (Fuijifilm, USA), which has 128 nozzels with a diameter of 50 μm. The print head is driven by two piezo elements, which can be driven separately. One piezo element drives the even and the other element the odd nozzle numbers. The distance between nozzle tip and hypothetical substrate in this study was set to 1.3 mm, which is a typical distance for binder jetting (Prasad and Smyth, 2016; Kwon, 2010).

##### 2.2.3.1 Printer preparation and cleaning

The printhead and ink reservoir were purged with at least 30 ml of ink to ensure that no residues of the previous liquid remained in the system before printing. After each purging step, the nozzle plate was cleaned using a fiber free cloth. After the printing process, the printhead was purged with 60 ml of suitable solvents to avoid polymer precipitation within the printhead. In cases of PVP and PVA, demineralized water of 80 °C was used. Before storage, the printhead and ink reservoir were purged with at least 60 ml of ethanol.

##### 2.2.3.2 Droplet characterization

Drop shape, drop velocity, and drop volume were analyzed using the integrated analysis program of the PIXDRO LP50 printer software. For this, two pictures were taken with the integrated high-speed camera with a defined delay and the program determined the droplet velocity from the traveled distance of the droplet center. The volume was determined via image analysis of the droplets. Pictures of the droplets were taken nine times within approximately 1 min for each setting and the data sets were processed using an inhouse developed Python (version 3.8.3) script to read out the droplet volume and speed determined by the printer software.

##### 2.2.3.3 Dwell time determination

For the determination of the optimal dwell time, the temperature was set to 30 °C, the voltage of both piezo elements to 90 V, and for the pulse shape a 1 μs rise and fall time was used. The dwell time was changed from 0 to 25 μs in 0.5 μs increments and the jetted droplets were analyzed nine times each.

##### 2.2.3.4 Jettability of inks

The jettability of inks was analyzed using the dwell time determined according to 2.2.3.3 and a fall and rising time of 1 μs. Both piezo elements were set to the same voltage, which was modified from 100 to 0 V in 2.5 V increments. Each setting was measured nine times. To assess the jettability, We and Ca numbers of the respective inks were calculated according to Eqs. (2), (4).

##### 2.2.3.5 Nozzle consistency

For the nozzle consistency the setting resulting in the highest droplet momentum, according to the jettability of inks (2.2.3.4), were used for each ink. The droplet volume for each ink was investigated on three separate days on the same nozzles distributed over the hole printhead. The jetted droplets were analyzed nine time for each run.

##### 2.2.3.6 Jetting stability

To investigate the jetting stability of the inks, the best performing PVP and PVA ink and 75% Glycerol as non-polymer containing standard were used. The inks were jetted, using the setting leading to the biggest droplet momentum, on three different days with a cleaning and storing procedure in between. The droplet volume was analyzed every 6 s, until the nozzle was not able to jet droplets, or the ink reservoir was empty. During the jetting no cleaning or wiping step was done.

## 3 Results and discussion

### 3.1 Jettability and dwell time optimization

To properly evaluate the results of the study, the jettability, i.e., the critical quality attributes for the resulting droplets, had to be defined. An ink was considered jettable when a single droplet was ejected out of the nozzle or satellite droplets and ligament were reabsorbed into a single droplet before hitting the hypothetical substrate in a distance of 1.3 mm (Fig. 2). Further, a minimum droplet speed needs to be defined based on the process demands. It has been reported that turbulent air flow might influence slower moving droplets more than faster ones, which could have a negative influence on the print accuracy. Higher movements speeds of the substrate under the printhead can induce turbulent air flow, which then effects small droplets leading to inaccurate printing (Hsiao et al., 2012; Rodriguez-Rivero et al., 2015). This may lead to defects within the layer and reduced print resolution, which might impact dosing accuracy, friability, and visual appearance of the dosage form and will have to be investigated in future studies. For the present study, a droplet speed of 3.0 m/s was set as minimum, as droplets with lower speed appeared to be more influenced by airflow during preliminary printing trials. This value has to be evaluated with proper binder jetting equipment, as a change of this criterion narrows or widens the jettable space.

After the definition of the jettability characteristics, the so-called jettable window and the optimal dwell time have to be defined to determine the limits in which an ink is considered jettable. For this, Nallan et al. defined the optimal dwell time as the shortest dwell time leading to droplets with the highest momentum to normalize the effect of the nozzle pressure (Nallan et al., 2014). Applying the same waveform on different ink systems results in different droplets speeds and volumes, as the pressure wave propagation is strongly dependent on the bulk modulus of the ink (Bogy and Talke, 1984). Another effect of utilizing the shortest possible dwell time with the highest momentum is the maximization of the jetting frequency, which will increased the absolute printing speed. Fig. 3 shows an exemplary graph of the dwell time optimization results of glycerol 75%. The same criterion for dwell time determination was used for all ink systems and for the binder containing inks. Optimal dwell times ranged from 6 μs to 10 μs. The results can be found in the supplementary materials (Table S1). The optimal dwell time can also be selected based on other process criteria if other attributes are more important, e.g., droplet velocity or droplet mass. In such cases, the dwell time can be adjusted accordingly via this approach.

### 3.2 Jettable window determination with solvent-based inks

The jettable window, the parameter space in which the previously selected criteria for jettability are met for the employed printhead was determined with pure solvents and solvents mixtures jetted with their optimal dwell time (Fig. 4 A). Only 75% glycerol, 62.5% glycerol, 50% glycerol and TGME displayed acceptable jettability (Table 3) indicating the existence of a jettable window with sufficient droplet speeds of 4.6 to 6.5 m/s between *Z*-numbers of 1.48 and 8.79. This is in line with literature where inks with Z-numbers between 1 and 10 are considered printable (Derby, 2011). For the other solvents, it was not possible to jet single droplets or only at relatively low droplet speeds (Glycerol 25% 1.45 m/s; isopropanol 1.66 m/s). The *Z*-numbers of the jettable window were further used as an indicator for the jettability of binder containing inks. It was expected that the jettable window would be similar for polymer containing solutions. Thus, polymer containing inks with Z-numbers between ∼1.5 and ∼ 8.8 should be jettable.

**Table 3:** Determination of the jettable window. Data shown with increasing Z-number. Droplet speed and volume shown as highest value for the generated single droplets. Droplet speed and volume (*n* = 9) are shown for the voltage resulting in the highest droplet momentum and viscosity (*n* = 3) is shown for the formulation. All data are shown as mean ± sd.

Solvent	Z-number	η [mPas]	Droplet velocity [m/s]	Droplet volume [pl]
Glycerol 75%	1.48	25.35 ± 0.25	5.62 ± 0.17	55.36 ± 0.43
Glycerol 62.5%	4.99	8.62 ± 0.12	6.53 ± 0.12	49.97 ± 0.47
TGME	6.89	5.95 ± 0.15	5.91 ± 0.21	53.23 ± 0.54
Glycerol 50%	8.79	4.61 ± 0.05	4.64 ± 0.02	25.14 ± 0.2
Glycerol 25%	22.09	1.69 ± 0.03	1.45 ± 0.13	47.56 ± 3.34
Isopropanol	17.04	1.66 ± 0.11	2.17 ± 0.02	12.68 ± 0.26
Tween 20 1%	41.82	0.84 ± 0.02	x	x

### 3.3 Jettable window determination with PVP-based inks

PVP K25 was chosen for its good water solubility over a wide temperature range (Guettari et al., 2017) and its intermediate molecular weight, which should allow higher binder contents in the ink. Inks containing 16.8–24 wt% PVP K25 were prepared. All investigated inks were jettable but the ink containing 16.8 wt% PVP K25 already indicates the lower boundary of the jettable window with a *Z*-number of 4.39 (Fig. 4 B). This can be derived from the small range where single droplets were formed and an increase in multiple satellite droplet formation at higher velocities. The decreased droplet velocity of the 24.00 wt% ink (2.57 ± 3.14 × 10^−3^ m/s) indicates that the upper border of the jettable window was close to a *Z*-number of 1.57, as the defined minimum speed was passed already (Fig. 4 B, Table 4).

**Table 4:** Jettability of PVP K25 inks with calculated Z-number. Droplet speed and droplet volume shown as highest value for generated single droplets. Speed and volume are shown as mean ± sd for *n* = 9. Viscosity values shown as mean ± sd for *n* = 3**.**

PVP K25 [wt%]	Z-number	*η* [mPa*s]	Droplet speed [m/s]	Droplet volume [pl]
16.80	4.39	8.59 ± 0.36	3.74 ± 0.00	32.6 ± 0.3
19.82	3.00	13.30 ± 0.16	5.12 ± 0.05	57.5 ± 0.4
21.94	2.30	17.68 ± 0.17	7.88 ± 0.31	66.7 ± 0.3
24.00	1.57	23.21 ± 0.62	2.57 ± 0.00	57.0 ± 0.5

**Fig. 5:**
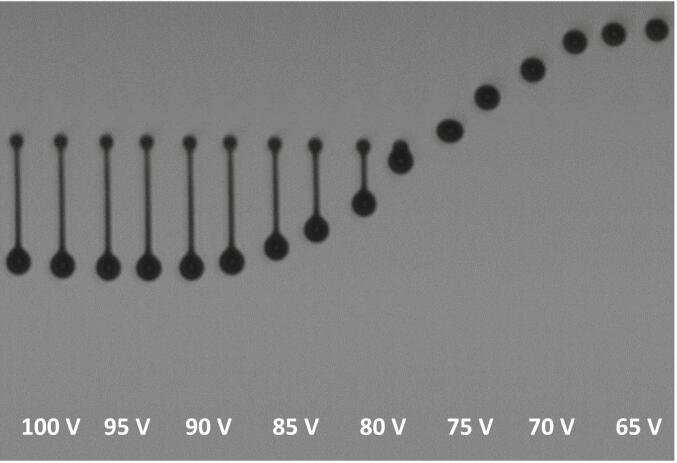
Jetting behavior of PVP K25 21.94 wt% ink at 30 °C jetted with t_dwell_ = 8.0 μs and same delay for each drop. Printhead voltage was changed from 100 to 62.5 V in 2.5 V increments. Lower border is at the powder bed height.

The jettable window of PVP K25 is consequently very narrow and ranges only from Z-numbers of 1.57 to 4.39. This is contradictory to literature as the jettable window was smaller than commonly assumed (1 < Z < 10). The ink containing 21.94 wt% PVP showed already a tendency to ligament formation at Ca numbers between 4.40 and 4.03 and We numbers of 102.66–86.14, which were all reabsorbed within the printing distance (Fig. 5). Considering that for binder jetting inks, higher binder concentrations are beneficial, this ink was found to have the best jettability as it has the highest polmer concentration with a droplet velocity > 3 m/s. And at lower binder contents the formation of single and multiple satellite droplets occurred.

Based on the defined criteria, the best results were achieved with PVP 21.94 wt% and a maximum excitation voltage of 97.5 V, which led to a droplet speed of 7.88 m/s and a volume of 66.7 pl, combining the highest droplets speed with highest droplet volume. The jettable window for PVP inks was consequently much smaller than the jettable windows of solvents and solvent mixture. The 75% glycerol ink had higher droplet velocities and more pronounced but reversing satellite droplet formation at a lower *Z*-number compared to PVP 24.00 wt% ink. This indicates that the upper limit of the jettable window of the 75% glycerol ink may not have been reached.

The reduction of the jettable space is very likely caused by the introduction of a viscoelastic polymer to the mostly Newtonian solvent system. The lower border of the jettable window of PVP K25 was reached already with a Z-number of 4.39 compared to Z-numbers of up to 8.79 for the solvents. This indicates that certain properties introduced by the PVP reduce the jettability of the ink. In literature, it is described that viscoelastic properties can drastically change the jetting behavior, often leading to a reduction of ink jettability (Hoath et al., 2012). It is reported that viscoelasticity is a major cause for ligament formation and can be the main driving force behind the break-up of the ligaments. Correlations between an increase in elasticity or molecular weight and the decrease of the break-up length have been published (de Gans et al., 2004; de Gans et al., 2005). The break-up length describes the distance between the point where the liquid jet breaks up into droplets and the nozzle. When the breakup length increases the ligament length increases until the point where the ligaments do not break (Hoath et al., 2014; Clasen et al., 2009). This can lead to so called “beads on a string”, where all jetted droplets are connected to the following droplet with a ligament, which can reduce the printing quality (Hoath et al., 2014; Clasen et al., 2009). For accurate printing, formed ligaments should be reabsorbed into the main droplet within the printing distance. At high viscoelastic forces the break-up can be hindered, which results in droplets that do not detach from the nozzle but are drawn back into the nozzle (de Gans et al., 2004; Clasen et al., 2012). In this study, we were not able to determine the viscoelastic properties of the employed inks as the frequency sweep at the linear viscoelastic region (LVER) resulted in non-interpretable data of low quality. Within the frame of the study we could not identify a reason for the failed measurements.

### 3.4 Jettable window determination with PVA-based inks

**Fig. 6:**
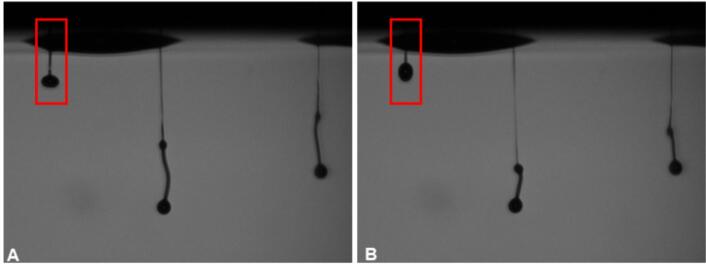
Jetting of PVA 18–88 2.75 wt% ink at nozzle plate. In the red box a droplet is shown that was reabsorbed by the nozzle plate after initial ejection, which led to the nozzle plate wetting. B was recorded with a delay of 10 μs after A. (For interpretation of the references to colour in this figure legend, the reader is referred to the web version of this article.)

Two different qualities of PVA, PVA 18–88 and PVA 4–88, were chosen for the next part of the study, as PVA is widely used in the pharmaceutical field. PVA is soluble in water and mainly insoluble in organic solvents, which might limit the use of organic solvents to modify the drying time and coffee stain effect. The coffee stain effect describes the effect that after drying the edge of the residue will have a higher solid content compared to the inner part (Deegan et al., 1997). The first number in the name of the PVA quality denotes for the mean viscosity in mPa*s of a 4 wt% aqueous solution. The second number represents the degree of hydrolyzation of the acetyl groups in %. PVA 18–88 is consequently the higher viscous quality of PVA. Aqueous polymer solutions of PVA 18–88 with *Z*-numbers in the jettable window identified with solvent inks were prepared. Only the ink with a low PVA 18–88 content of 2.75 wt% led to the ejection of any droplets (Fig. 6). Higher concentrations resulted in inks which were not jettable. The ink with the lowest polymer content of 2.75 wt% already had a Z-number of 6.46, which is located at the lower boarder of the jettable window explored using solvents. The ejected droplets showed formation of small ligaments and an inconsistent ejection out of the nozzle. Some ligaments were reabsorbed by the nozzle plate after ejection, leading to severe nozzle plate wetting and a buildup of ink (Fig. 6, red box). This further indicates that polymer specific properties, such as viscoelastic behavior, might have a high impact on the jettability of ink and that the jettable window can shift drastically depending on the polymer properties.

To increase the concentration of binder in the ink, PVA 4–88 with a lower molecular mass and viscosity was chosen. Higher binder contents are expected to be beneficial for binder jetting, as this results in more solid bridges within the printed dosage forms, leading to higher tensile strength and lower friability. Inks with a PVA 4–88 content of up to 9.43 wt% were jettable (Fig. 4 C), which showed that the upper border of the jettable window for PVA 4–88 is between *Z*-numbers of 2.00 and 2.41. This further demonstrates the massive influence of the choice of polymer on the jettable window. PVP inks with a Z-number of 1.57 still led to single droplet formation while PVA 4–88 was not jettable at a *Z*-number of 2.00 and below. The jettable window of PVA 4–88 inks was only between Z-numbers of 2.41 and 4.33 (Table 5). With higher Z-numbers the satellite droplet formation increased and a high polymer content was found to be favorable.

**Table 5:** PVA 4–88 inks with calculated Z-number. Inks displayed in red where not jettable. Droplet speed and droplet volume shown as highest value for the generated single droplet. Speed and volume are shown as mean ± sd for *n* = 9. Viscosity values shown as mean ± sd for *n* = 3.

PVA 4–88 [wt%]	*Z*-number	*η* [mPa*s]	Droplet speed [m/s]	Droplet volume [pl]
7.51	4.33	9.58 ± 0.02	6.57 ± 0.19	65.1 ± 0.5
8.27	3.42	11.93 ± 0.21	6.25 ± 0.17	59.2 ± 0.6
8.90	2.81	14.58 ± 0.15	3.13 ± 0.02	59.7 ± 0.5
9.43	2.41	17.06 ± 1.04	2.21 ± 0.1	58.7 ± 0.3
9.89	2.00	20.34 ± 0.96	X	X

This data points out the limitations of the general assumption that jettability is given between Z-numbers of 1–10. This might be used as an indicator for early trials to investigate the jettable window of a given printhead but is not sufficient for proper ink development. As shown here, the addition of dissolved polymers that introduce non-Newtonian rheological properties can result in a severe shift or reduction in the jettable window. On the other hand, our data shows that the jettability of pure solvents is closer to the suggested Z-number range. The effect of added APIs is likely negligible compared to the impact of polymer and surfactant on viscosity, density and surface tension. The effect of API addition on non-Newtonian rheological properties would be highly dependent on API-polymer interaction and might differ for every API. Nonetheless, the effect of other dissolved materials, such as APIs, would need to be further investigated.

### 3.5 Printing consistency and long-term performance

**Fig. 7:**
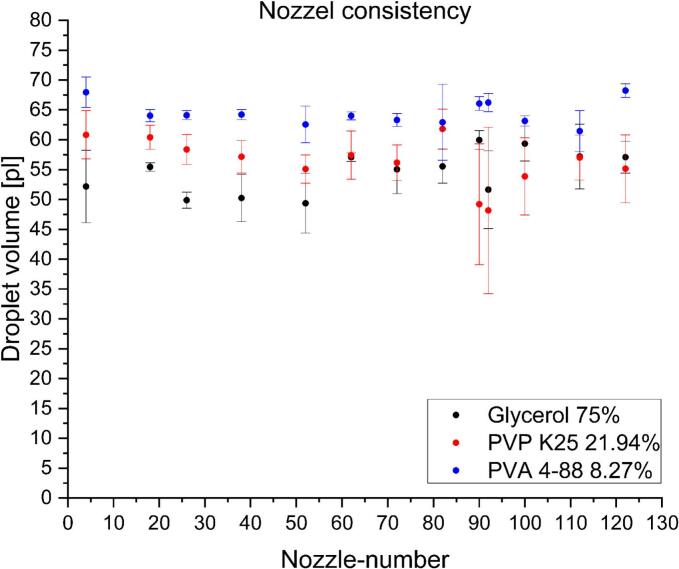
Consistency of generated droplets over the printhead with pure solvent, PVP and PVA ink, with the best droplet properties (mean ± SD, n = 9).

To better understand the ink performance during the printing process, the consistency of the droplet generation from individual nozzles of the print head and the stability of the jetting process over time were investigated. These factors should also be considered for the choice of the ink candidate during development. The consistency of the droplet generation over the printhead was determined for best performing pure solvent ink, glycerol 75%, and best-performing polymer-based inks, PVP K25 21.94% and PVA 4–88 8.27% (Fig. 7).

The PVA 4–88 containing ink showed the best droplet consistency across all 128 nozzles of the employed printhead. The ink pattern had less fluctuations between the nozzles and the printing runs. For the printing process, a pronounced difference in the generated droplet volume over the printhead can lead to defects in the layer which might influence dosage form properties. If not only the binder but in addition the API would be dissolved in the ink, failing nozzles or variations in nozzle consistency will lead to a decrease in the applied volume of ink in resulting dosage form and compromising content uniformity.

**Fig. 8:**
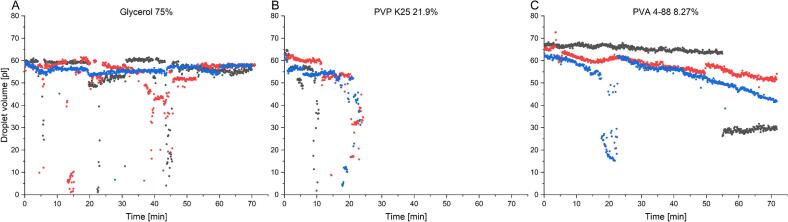
Jetting stability over time of different inks printed at 1000 Hz using the optimal dwell time. The different colors indicate different runs for a working nozzle.

As dosage forms consist of several layers, the jetting behavior over time is also an important ink and process characteristic. During printing processes, the nozzle plate can also be wetted with ink, which can lead to a reduction in droplet size or to nozzle blockage. This can impact the printing process and extensive cleaning cycles would be necessary, increasing processing time and ink wastage. To this end, the jetting behavior over time was investigated for the best binder-containing inks and the pure solvent ink as a reference until the nozzle clogged or the ink reservoir was empty, without interfering in the printing process (Fig. 8).

**Fig. 9:**
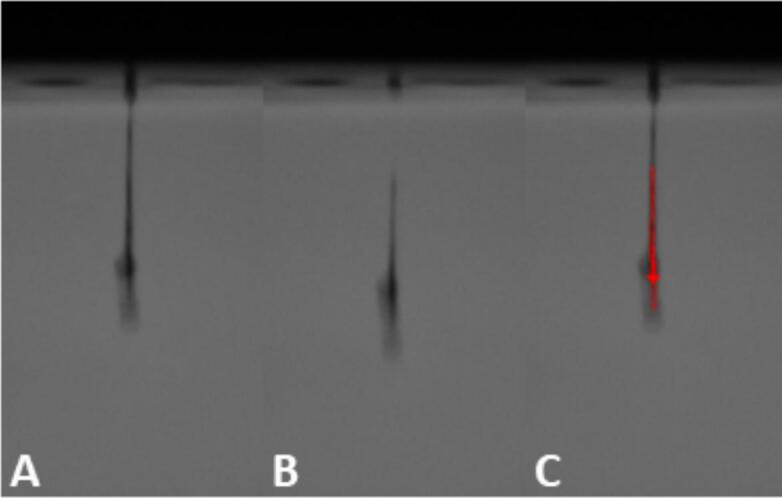
Droplets that move out of the focus in the z axes, likely caused by a clogged or over wetted nozzle. A) picture 1, B) picture 2 with a delay of 5 μs to picture 1. C merged picture of 1 and 2 for the automated determination of droplet volume and speed by the software from the Pixdro printer.

Glycerol 75% was jettable until no ink was left without pronounced fluctuations of the droplet volume. The nozzle neither clogged nor was a drastic, prolonged drop in droplet volume observable. The sudden decreases of the droplet volume visible in Fig. 8 A can be attributed to the automated volume determination by the software. Sometimes, droplets would move out of focus of the high speed camera and the volume calculation was inaccurate (Fig. 9). This effect was further responsible for the sudden drop in droplet volume for the PVA 4–88 ink at around 20 min (Fig. 8 C, bule dots). The reason for the movement of droplets out of the camera focus may be caused by over-wetted or partially clogged nozzles. While this can also lead to different droplet volumes, this could not be analyzed with the experimental setup at hand.

PVA 4–88 ink showed a better process stability compared to the PVP K25 ink, where the jetting process stopped due to nozzle clogging after 15 to 20 min. The introduction of viscoelastic properties may increase the nozzle plate wetting caused by the return of not detached droplets to the plate. This could lead to a reduction in printing stability as the tendency of nozzle clogging is increased by an overwetted nozzle plate. If this is the case, PVA containing ink should have less pronounced viscoelastic properties compared to PVP. Further, the difference in viscosity could also have an influence on the nozzle clogging, as micro air bubbles might build up more easily within the printhead for higher viscous inks leading to nozzle plate wetting and nozzle clogging (de Jong et al., 2006). During the jetting of PVA 4–88 the droplet volume of one run (Fig. 8, C grey dots) decreased by approximately 50% until the end of the jetting process. This decrease was not the result of droplets moving out of focus. Here the decreased droplet volume might be caused by an overwetted or slightly clogged nozzle, due to the polymer content. Nozzel clogging or over wetting might led to the constant decrease in droplet volume for PVA and PVP inks during the run. As compared to the Glycerol ink where no trend was observable. Overall, it is observable that the introduction of polymer reduced the jetting stability of the droplet volume compared to the pure solvent reference ink. Based on these observations, the PVA 4–88 ink is the favorable ink candidate for further developments.

## 4 Conclusion

The ink formulation approach of Nallan et al. was successfully transferred to pharmaceutical inkjet printing. This approach allows a systematic and robust formulation and process development and optimization based on reliable measurements. This presents a significant advantage over previous approaches that were solely based on literature data or requirements published by printhead manufacturers. While we expected that the general assumption that inks with *Z*-numbers ranging from 1 to 10 result in jettable inks cannot be generally true, we were surprised to see that, especially when polymers were added, the Z-number ranges could be considerably smaller. This of course also depends on the initial definition of what is deemed jettable. The introduction of viscoelastic materials results in complications as additional failure modes can occur, e.g., the reabsorption of droplets to the nozzle plate.

With the presented approach, inks can be designed for specific processes based on a systematic understanding of the jetting behavior. The jettable window enables formulation scientists to identify if an ink will be still jettable after a composition change based on its change in viscosity, density, or surface tension. The viscosity is proportionally linked to the We-number as the Ca-number is to the density, whereas the surface tension is anti-proportionally linked to both, the We and Ca-number. These parameters could be used to calculate whether an ink is jettable or not based on the jettable window without the need for experimental trials in the future. Also, not only the jetted droplets should be considered for the ink design and the choice of ink candidate. Process stability is also a parameter that should be considered for the ink development, as the stability of the jetting process might have a high impact of the overall tablet quality and production time.

In this study, we did not investigate the addition of suspended particles, which further increases the risk of nozzle blockage. Also additional dissolved substances such as API or polymers can also have a high effect on the process stability by increasing the risk of nozzle blockage by precipitating API or polymer at the nozzle plate. An overwetted nozzle plate, which can result in the inactivation or blockage of nozzles, should be avoided as good as possible. Our data suggests that consistent printing performance could prolong the time between cleaning cycles, leading to a more efficient production. The investigation of potential nozzle cleaning was not within the scope of the present study. Furthermore, in depth investigations into the impact of viscoelastic properties of pharmaceutical polymers should be conducted in future studies.

## CRediT authorship contribution statement

**Maximilian Schulz:** Writing – review & editing, Writing – original draft, Visualization, Software, Methodology, Investigation, Formal analysis, Data curation, Conceptualization. **Malte Bogdahn:** Writing – review & editing, Conceptualization. **Simon Geissler:** Writing – review & editing, Conceptualization. **Julian Quodbach:** Writing – review & editing, Supervision, Project administration, Methodology, Conceptualization.

## Declaration of competing interest

The authors declare that they have no known competing financial interests or personal relationships that could have appeared to influence the work reported in this paper.

## Data Availability

Data will be made available on request.

## Appendix A Supplementary data

Supplementary material 1 Image 1

Supplementary material 2 Image 2
