# Supplementary material for: Transfer of a rational formulation and process development approach for 2D inks for pharmaceutical 2D and 3D printing
Source: Int J Pharm X. 2024 May 10;7:100256. doi: 10.1016/j.ijpx.2024.100256 (PMC11176655; doi:10.1016/j.ijpx.2024.100256)
Supplement: Supplementary file 2 — Supplementary material 2 [file mmc2.docx]

***Supplementary information***

**Transfer of a rational formulation and process development approach for 2D inks for pharmaceutical 2D and 3D printing**

**Maximilian Schulz^a^, Malte Bogdahn^b^, Simon Geissler^b^, Julian Quodbach^a,c,*^**

^a^Institute of Pharmaceutics and Biopharmaceutics, Heinrich Heine University Düsseldorf, Universitätsstr. 1, Düsseldorf, Germany

^b^Merck Healthcare KGaA, Frankfurter Str. 250, Darmstadt, Germany

^c^Division of Pharmaceutics, Utrecht Institute for Pharmaceutical Sciences, Utrecht University,
Universiteitsweg 99, Utrecht, The Netherlands

Table S1: Optimal dwell times determined, set ink pressure, density and surface tension for all used inks.

| **Inks** | **Optimal dwell time [μs]** | **Ink pressure [mbar]** | **density [g/cm³]** | **surface tension [mN/m]** |
| --- | --- | --- | --- | --- |
| 75 % glycerol | 8.0 | -15.5 | 1.189 | 23.708 |
| 62.5 % glycerol | 7.0 | -15.5 | 1.157 | 31.966 |
| 50 % glycerol | 7.5 | -15.5 | 1.122 | 29.340 |
| 25 % glycerol | 8.0 | -15.5 | 1.056 | 26.390 |
| 1 % polysorbate 20 | 9.0 | -15.5 | 0.994 | 24.898 |
| Isopropanol | 6.0 | -20.5 | 0.775 | 20.660 |
| TGME | 7.5 | -19.0 | 1.009 | 33.269 |
| PVP K25 16.80 % | 9.0 | -15.5 | 1.032 | 27.558 |
| PVP K25 19.82 % | 8.0 | -15.5 | 1.039 | 30.557 |
| PVP K25 21.94 % | 8.0 | -15.5 | 1.044 | 31.701 |
| PVP K25 24.00 % | 8.5 | -15.5 | 1.049 | 25.248 |
| PVA 4-88 7.51 % | 10.0 | -15.5 | 1.014 | 33.317 |
| PVA 4-88 8.27 % | 7.5 | -15.5 | 1.016 | 32.812 |
| PVA 4-88 8.90 % | 7.5 | -15.5 | 1.018 | 33.084 |
| PVA 4-88 9.43 % | 7.5 | -15.5 | 1.019 | 33.100 |
| PVP 19.82 % + 5 % ethanol | 8.5 | -15.5 | 1.009 | 33.294 |
| PVP 19.82 % + 10 % ethanol | 8.5 | -15.5 | 0.997 | 33.261 |
| PVA 4-88 8.27 % + 5 % ethanol | 8.5 | -15.5 | 1.033 | 31.811 |
| PVA 4-88 8.27 % + 10 % ethanol | 8.0 | -15.5 | 1.025 | 32.516 |
